# 

**Published:** 1850-03

**Authors:** 


### WESTERN JOURNAL

OF

### MEDICINE AND SURGERY.

### EDITORS.

LUNSFORD P. YANDELL, M. D.

PROFESSOR OF PHYSIOLOGY AND PATHOLOGICAL ANATOMY IN THE UNIVERSITY OF LOUISVILLE.

AND

THEODORE S. BELL, M.D.

### DELINQUENT SUBSCRIBERS.

This numerous class must see the necessity of making prompt remittances. We furnish a Journal which is not only a record of the progress of all the departments of Practical Medicine, but which contains the principles of practice adapted to the West and South. Its publication is attended with a heavy expenditure, and the subscribers should enable us to meet this expenditure without any trouble. We feel every assurance that we give them their money's worth and we hope they will promptly respond to this call by remitting their dues.

PRENTICE & WEISSINGER, *Publishers*

### *Receipts of Medical Journal for Feb.* 1850.

| | |
|---|---|
| Dr. L. S. Tolson, Magee's Bridge, Miss. | $5 00 |
| H. W. Dickson, Portersville, Tenn., | 5 00 |
| J. N. Lewis, Jefferson county, Ky., | 5 00 |
| W. D. Rodes, Rocky Hill, Ky.. | 5 00 |
| E. T. Collett, Fort Des Moins, Iowa, | 5 00 |
| B. F. Summers, Shepherdsville, Ky., | 5 00 |
| J. R. Thomson, North Salem, Ind., | 5 00 |
| J. W. S. Frierson, Columbia, Tenn.. | 2 00 |
| W. Stapp. Lewisport, Ky., | 5 00 |
| J. J. Miles, Charleston, Ill., | 11 40 |
| J. S. Croutch, Clarksville, Tenn., | 5 00 |
| Debruler & Crooks, Rockport, Ind. | 5 00 |
| S. C. Gerin, Shongalo, Miss., | 5 00 |
| J. F. Booth, Fulton, Miss., | 5 00 |

The WESTERN JOURNAL OF MEDICINE and SURGERY is published monthly by the undersigned, at the corner of Main and Fifth streets, Louisville, at $5 per annum, payable in advance. Each number contains 96 pages, making two volumes in the year of more than 550 pages each.

Contributions to its pages by the Physicians of the Valley of the Mississippi addressed tothe Editors, are respectfully solicited.

Letters on business, to be addressed, postage paid, to the publishers.

Jauuary, 1844. PRENTICE & WEISSINGER.

